# Lessons Learned from the Development and Roll-Out of the rVSVΔG-ZEBOV-GP *Zaire ebolavirus* Vaccine to Inform Marburg Virus and *Sudan ebolavirus* Vaccines

**DOI:** 10.3390/vaccines10091446

**Published:** 2022-09-01

**Authors:** Beth-Ann G. Coller, William Lapps, Mahum Yunus, Samantha Bruno, Michael J. Eichberg, Andrew Wen-Tseng Lee, Kenneth Liu, Rosybel Drury, Jules Millogo, Louis Robert Macareo, Thomas H. Armstrong, Jeffrey T. Blue, Lynne A. Isopi, Melissa Hughes, Susan M. VanRheenen, Jonathan Deutsch, Joan G. Tell, Sheri A. Dubey

**Affiliations:** 1 Merck & Co., Inc., Rahway, NJ 07065, USA; 2 MSD, 69007 Lyon, France

**Keywords:** rVSV, recombinant vaccine, vesicular stomatitis virus, *Zaire ebolavirus*, Marburg virus, *Sudan ebolavirus*, ERVEBO^®^

## Abstract

This review describes key aspects of the development of the rVSVΔG-ZEBOV-GP Ebola vaccine and key activities which are continuing to further expand our knowledge of the product. Extensive partnerships and innovative approaches were used to address the various challenges encountered during this process. The rVSVΔG-ZEBOV-GP Ebola vaccine was initially approved by the European Medicines Agency and prequalified by the World Health Organization in November 2019. It was approved by the United States Food and Drug Administration in December 2019 and approved in five African countries within 90 days of prequalification. The development resulted in the first stockpile of a registered Ebola vaccine that is available to support outbreak response. This also provides insights into how the example of rVSVΔG-ZEBOV-GP can inform the development of vaccines for *Sudan ebolavirus*, Marburg virus, and other emerging epidemic diseases in terms of the types of approaches and data needed to support product registration, availability, and the use of a filovirus vaccine.

## 1. Introduction

Filoviruses represent an ongoing threat to human and animal health, and the development of safe and effective vaccines to prevent disease linked to these pathogens is an important effort. Beginning in late 2013, the emergence of *Zaire ebolavirus* (EBOV) in West Africa resulted in the largest filovirus outbreak recorded to date with more than 28,000 cases and 11,000 deaths [1]. The catastrophic impact of this West African outbreak and the potential for global spread catalyzed major efforts to develop vaccine and therapeutic countermeasures to EBOV and other related filoviruses. This effort included the accelerated development of a recombinant vesicular stomatitis virus-based vaccine, rVSVΔG-ZEBOV-GP (V920, trade name ERVEBO^®^), which had initially been constructed and characterized by scientists at the Public Health Agency of Canada (PHAC) as an anti-bioterrorism agent [2]. The development of rVSVΔG-ZEBOV-GP provides an important example of how vaccines for epidemic outbreaks can be developed through partnerships and creative approaches to address clinical and regulatory requirements. This Ebola vaccine is now registered in more than 40 countries, including the U.S. [3], EU [4], Switzerland, UK, and numerous countries in Africa, and a global stockpile is available to support individuals at risk of exposure. The aim of this review was to highlight learnings from the development and roll-out of rVSVΔG-ZEBOV-GP applicable to the development of other vaccines. This review describes the key aspects of the clinical and non-clinical development of rVSVΔG-ZEBOV-GP pre-and post-product registration, regulatory approval, its use in Ebola outbreak response, manufacturing and stockpiling considerations, recommendations for its use, and environmental risk assessment. These key activities are continuing to further expand our knowledge of the product and provide insights into how the example of rVSVΔG-ZEBOV-GP can inform the development of vaccines for *Sudan ebolavirus*, Marburg virus, and other emerging epidemic diseases.

## 2. Clinical and Non-Clinical Development of rVSVΔG-ZEBOV-GP Pre- and Post-Product Registration

### 2.1. Early Characterization and Clinical Development Leading to Registration of rVSVΔG-ZEBOV-GP

Pivotal to the successful and rapid development of rVSVΔG-ZEBOV-GP was the characterization, manufacturing, and early development work done by PHAC. The team in the National Microbiology Laboratory at PHAC developed the construct and conducted the early animal studies suggesting that the construct had significant potential as a vaccine candidate [2]. Based upon that promising work, they had the foresight to develop a current Good Manufacturing Practice (cGMP) compliant manufacturing process so that material that could be used in human subjects would be available. PHAC, as well as the U.S. Department of Defense, contracted with an experienced manufacturing partner, IDT Biologika (Dessau-Rosslau, Germany), to manufacture cGMP clinical supplies and those materials were pivotal to enabling the early launch of Phase 1 clinical trials towards the end of 2014 when the West African outbreak was at its peak. Their foresight and planning were key to successfully advancing the vaccine candidate quickly in the context of the outbreak, highlighting the importance of conducting basic research and preclinical development in “peace time” to be prepared to move quickly when an outbreak occurs.

Phase 1 clinical trials began in October 2014 and included eight Phase 1 trials (V920-001 to V920-008) conducted in the U.S., Canada, Switzerland, Germany, Gabon, and Kenya [5,6,7,8,9,10,11]. Data from the Phase 1 trials informed a decision on the dose of vaccine to be advanced into Phase 2 and 3 clinical trials, the first of which began in February 2015, just 4 months after the start of Phase 1 trials. The Phase 2/3 program that supported registration of rVSVΔG-ZEBOV-GP is an example of diverse and creative study designs that demonstrated the power of partnership to advance public health priorities in an emergency. Four Phase 2/3 trials formed the basis of the data to support the registration of rVSVΔG-ZEBOV-GP: PREVAIL 1 trial (V920-009; NCT02344407) conducted in Liberia and sponsored by the U.S. National Institutes of Health (NIH) [12]; Ebola Ça Suffit! trial (V920-010; PACTR201503001057193) conducted in Guinea and sponsored by the World Health Organization (WHO) [13]; STRIVE trial (V920-011; NCT02378753; PACTR201502001037220) conducted in Sierra Leone and sponsored by the U.S. Centers for Disease Control and Prevention [14]; and the safety and lot consistency trial (V920-012; NCT02503202) conducted in the U.S., Spain, and Canada and sponsored by Merck Sharp & Dohme LLC, a subsidiary of Merck & Co., Inc, Rahway, NJ, USA (MSD) [15,16]. Each of these studies had a unique design and incorporated best practices from the broad clinical research community, as well as specific feedback from the communities where the studies were conducted. The engagement of the national and local communities and leadership was critical to ensure that the studies aligned with local customs and that there was a clear understanding in the community and by the individual participants about the purpose of the study and how the data would be used (e.g., PREVAIL 1 [12]) [17]. The application of ring vaccination as a clinical trial approach [18] was particularly innovative and enabled an assessment of efficacy despite the significant drop in Ebola virus disease (EVD) cases by early 2015 when the Phase 2/3 studies started [13]. Cumulatively, the Phase 1, 2, and 3 clinical trials provided data from approximately 16,000 participants, including safety, efficacy, and immunogenicity data that supported the registration of rVSVΔG-ZEBOV-GP beginning in November 2019 [19].

Early non-human primate (NHP) studies conducted by PHAC assessed vaccine-induced immunogenicity and efficacy. Collectively, these studies demonstrated robust antibody titers and high levels of protection from EBOV challenge administered at time of peak immune response, 28 days after a single vaccine administration [20,21,22,23]. In parallel to the clinical development, the additional non-clinical development of rVSVΔG-ZEBOV-GP was conducted with the support of, and through collaboration with, the U.S. Department of Defense. These NHP studies confirmed the robust antibody responses and protection from challenge 42 days post-vaccination across a wide range of doses [24,25]. Full protection in as few as 7 days post-vaccination and partial protection at 3 days post-vaccination have been reported in this stringent NHP model [26]. The NHP data, together with clinical efficacy starting at 10 days post-vaccination [13], support use of a single rVSVΔG-ZEBOV-GP vaccination in an outbreak setting where the induction of rapid protection is imperative. Additional non-clinical development included mouse and NHP repeat dose toxicity studies [25], a biodistribution study in NHPs [25], and studies related to the potential for environmental risk linked to the fact that rVSVΔG-ZEBOV-GP is a genetically modified organism [27]. Further discussion of this work and the recent advancements is provided in later sections.

### 2.2. Post-Product Registration Advancements in Non-Clinical and Clinical Development

**Figure 1:**
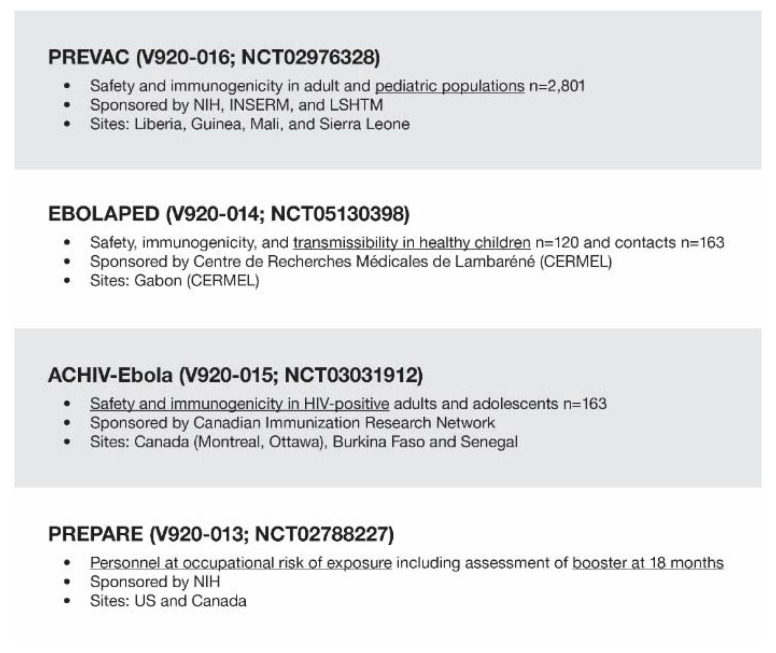
Ongoing clinical trial work to fill data gaps: ongoing partnerships. INSERM, Institut National de la Santé et de la Recherche Médicale; LSHTM, London School of Hygiene & Tropical Medicine; NIH, National Institutes of Health; U.S., United States.

While the clinical data set to support product registration was robust, additional clinical work (Figure 1) has continued to further the understanding of the performance of rVSVΔG-ZEBOV-GP in additional populations (pediatric populations, HIV-positive individuals) to further explore vaccine virus shedding in these populations and better understand how immunogenicity and protection are related. Ongoing data collection to expand our understanding of the durability of immunogenicity and protection is also important to inform recommendations on the use of the product in outbreaks and in more prospective use in advance of outbreaks.

#### 2.2.1. Pediatric Populations

Following the 2014–2016 EVD emergency in Western Africa, the continued risk for additional outbreaks warranted the evaluation of rVSVΔG-ZEBOV-GP in pediatric populations as important at-risk groups for EVD. A Phase 1 randomized, dose-ranging, open-label trial (V920-007; PACTR201411000919191) was conducted to evaluate the safety and immunogenicity of rVSVΔG-ZEBOV-GP in healthy children aged 6–17 years and adults living in Gabon [10].

To follow up on and extend the pediatric results in the Gabon trial [10], a cooperative Phase 2 study (PREVAC; V920-016; NCT02876328) enrolled 1400 adults and 1401 children ≥1 year of age to receive rVSVΔG-ZEBOV-GP, with and without a booster dose or placebo [28]. The study was conducted in Guinea, Liberia, Mali, and Sierra Leone under the sponsorship of the National Institutes of Health (NIH), Bethesda, MD, USA; Institut National de la Santé et de la Recherche Médicale (INSERM), Paris, France; and London School of Hygiene & Tropical Medicine (LSHTM), London, UK, with MSD providing the rVSVΔG-ZEBOV-GP vaccine supply, validated immunogenicity laboratory testing, and clinical operations support [28]. The study demonstrated that both children and adults had superior immune responses to one or two doses of rVSVΔG-ZEBOV-GP, as compared to placebo, at 12 months after vaccination [29]. Children had non-inferior immune responses to vaccine as compared to adults. Both one- and two-dose regimens of rVSVΔG-ZEBOV-GP showed similar rates of serious adverse events and deaths as compared to placebo, and solicited injection-site and systemic reactions were consistent with the known safety profile in children and adults [30]. Data from validated immunogenicity assays and data related to the shedding of vaccine virus in children are anticipated in the coming months.

In addition, a study is being conducted at the Centre de Recherches Médicale de Lambaréné (CERMEL), Lambaréné, Gabon, as part of the EBOPLUS Consortium supported by the Innovative Medicines Initiative (IMI) (EBOLAPED; V920-014; NCT05130398). This study specifically focuses on the generation of additional data related to viral shedding in children and the potential for transmission of vaccine virus to contacts of vaccinees.

#### 2.2.2. HIV-Positive Individuals

Given that future Ebola outbreaks are likely to occur in countries where HIV infection is endemic or present in large segments of the population, it is important to evaluate the safety and immunogenicity of the rVSVΔG-ZEBOV-GP vaccine in the HIV-positive population. Epidemic responses, including vaccination roll-out efforts, are urgent by necessity and HIV testing may not be possible. A randomized, placebo-controlled Phase 2 study (ACHIV-Ebola; V920-015; NCT03031912) sponsored by the Canadian Immunization Research Network was initiated to evaluate the safety, tolerability, and immunogenicity of rVSVΔG-ZEBOV-GP vaccine in HIV-infected adults and adolescents in Canada, Burkina Faso, and Senegal. Approximately 250 participants have been enrolled in a CD4-T cell count step-down design.

#### 2.2.3. Impact of Booster Doses

The need for booster doses of the rVSVΔG-ZEBOV-GP vaccine has not yet been established. The Phase 1 trial conducted by the NIH [5] and the PREVAC study [28] showed that a second dose of rVSVΔG-ZEBOV-GP vaccine given at 28 or 56 days, respectively, after the first dose was well tolerated, with generally lower incidences of solicited local and systemic adverse events after the second dose as compared to the first dose [5,30]. The second dose was immunogenic, boosting antibody titers 1 month after the second dose, although the boost was not sustained, with similar levels of glycoprotein-enzyme-linked immunosorbent assay (GP-ELISA) geometric mean antibody concentrations and seroresponse rates between the one- and two-dose regimens by month 12 [29]. An NIH-sponsored Phase 2, randomized, open-label study (PREPARE; V920-013; NCT02788227) is assessing the possible benefit of a booster (compared with no booster) when given at 18 months in individuals at occupational risk for EVD exposure. The study is being conducted in the U.S. and Canada, with the aim to evaluate the safety and immunogenicity of a booster dose given at this later time point.

#### 2.2.4. Human Studies Assessing Durability of Immunogenicity and Correlates of Protection

The initial demonstration of efficacy conducted in the Ebola Ça Suffit! trial [13] followed up subjects for 84 days and therefore long-term protection was not assessed. Based upon that limitation, efforts have continued to understand the durability of immunogenicity and to evaluate the relationship between immunogenicity and protection in human subjects. The durability of antibody titers as measured in validated GP-ELISA and plaque reduction neutralization test (PRNT) assays, was demonstrated for up to 2 years post-vaccination of a single dose of rVSVΔG-ZEBOV-GP in the MSD-sponsored safety and lot consistency study [16]. The durability of immunogenicity is being followed up for 5 years in the PREVAIL 1 [12] and PREVAC trials [28]. NHP models are also being leveraged to understand the durability of immunogenicity and protection as described in the next section.

The evaluation of the correlates of protection and immune response was performed at the population level using subjects vaccinated in Guinea, Liberia, and Sierra Leone with samples tested in the validated assays [31]. This post hoc analysis of potential correlates of protection demonstrated that for rVSVΔG-ZEBOV-GP, response patterns were similar for the GP-ELISA and the PRNT assay, with the ELISA titer providing a statistical correlate of protection at the population level. However, the analysis did not yield a consistent protective threshold that can be applied at the individual subject level to predict whether an individual is protected over time [31].

#### 2.2.5. NHP Studies Assessing Correlates and Durability of Protection

To facilitate immunobridging between NHP and human studies, the same validated EBOV GP-specific ELISA and PRNT were implemented in the rVSVΔG-ZEBOV-GP clinical and non-clinical program. The ELISA assay was developed by the Filovirus Animal Non-Clinical Group (FANG) [32] and was initially validated for human sample testing in several laboratories in support of clinical studies conducted by multiple vaccine developers [33]. In a collaboration between the U.S. Department of Defense, vaccine developers, and testing laboratories, the validated human ELISA was assessed for testing NHP samples and was validated for NHP serum upon demonstration that it provided comparable performance with both serum sources [34]. Separately, the PRNT assay developed for the rVSVΔG-ZEBOV-GP program is a species-neutral assay that measures antibody-induced neutralization of the vaccine virus in vitro and was validated for testing of both human and NHP samples [6,16].

In NHP studies with rVSVΔG-ZEBOV-GP, robust antibody titers and high protection levels upon EBOV challenge make it difficult to identify a minimum protective threshold [31]. Similar to the clinical analysis, the wider range of ELISA titers in NHP provided better differentiation for the assessment of a correlate of protection than PRNT [31]. However, NHP antibody levels after a single dose of rVSVΔG-ZEBOV-GP, tested in the same validated assay as the human samples, are typically much higher than in humans, up to approximately 10–20-fold higher, and therefore protective threshold levels in NHP could not be directly extrapolated to humans [31]. The post hoc analysis concluded that a dichotomous correlate of protection, also referred to as vaccine “take”, may be the most appropriate approach for this vaccine. A seroresponse in the validated GP-ELISA, defined as a post-vaccination immune response of ≥200 EU/mL and at least a 2-fold increase pre-to post-vaccination [35], was shown to be associated with protection [31]. This situation is analogous to smallpox, for which vaccine “take”, rather than any specific titer, is the accepted predictor of vaccine efficacy [36].

Regarding the durability of protection in NHPs, a high level of protection was maintained in NHPs that were challenged 4 months after receiving one or two doses of rVSVΔG-ZEBOV-GP vaccine administered at the human clinical dose level 60 days apart (unpublished data). A study to assess vaccine efficacy at 8 and 12 months post-vaccination is ongoing. The analyses of additional measures of immunogenicity, including Fc effector function, antibody isotype, antibody affinity, and T-cell responses, are also in progress in the ongoing NHP studies to assess potential alternative correlates of protection and potential changes to correlates over time.

### 2.3. Bridging to Marburg and Sudan Vaccine Viruses

**Figure 2:**
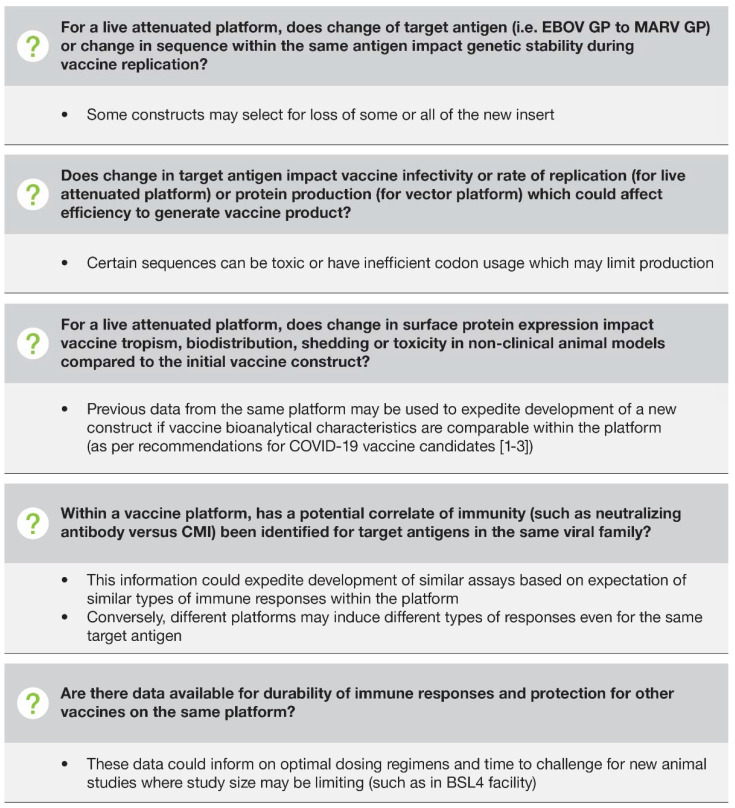
Key questions for new vaccine constructs based on shared platforms. BSL4, biosafety level 4; CMI, cell-mediated immunity; EBOV, Zaire ebolavirus; GP, glycoprotein; MARV, Marburg virus.

The clinical and non-clinical results for registered products, such as rVSVΔG-ZEBOV-GP, which are based on platform technologies, offer an attractive potential pathway for developing new vaccines for other filoviruses based on similar platform technologies. The demonstration of robust immunogenicity in humans, and efficacy in relevant animal models for additional targets that utilize the same expression platform, would suggest a high probability of success for such vaccine candidates. Demonstration that the immune responses induced to other targets expressed in the same platform including innate, humoral, and cellular immunity are similar to the proven target would provide confidence that similar protection may also be achieved. As highlighted in the prior section, it is important to establish validated clinical assays that can be used both in human sample testing and animal testing to characterize novel vaccine targets. Utilizing such assays with proven performance enables the results from testing to be compared and provides information on the best construct or dosage necessary for success in studies. However, bridging directly to another vaccine that is engineered with a new antigen needs careful evaluation. If significantly different in structure and function, a new antigen may show targeting to different tissues and have different effects on safety and efficacy. In addition, determination of the proper dose needs to be carried out empirically. Therefore, bridging needs to be informed by the evaluation of safety, immunogenicity, and efficacy, utilizing both non-clinical and clinical studies to demonstrate the effect of the new construct. Key questions to answer for new vaccine constructs are summarized in Figure 2 [37,38,39].

## 3. Regulatory Aspects

**Figure 3:**
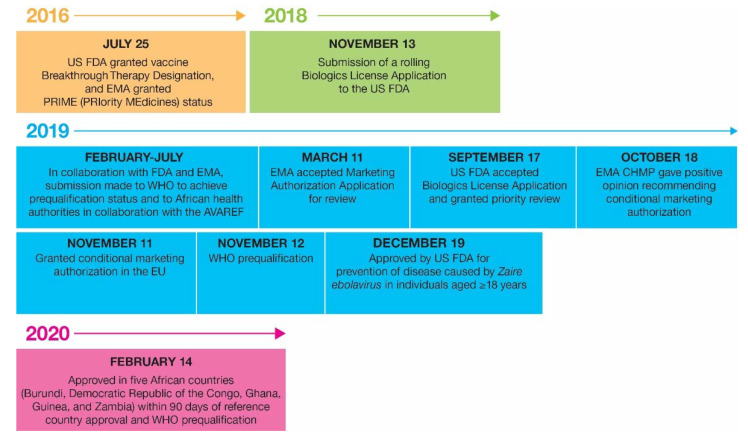
Regulatory timeline for rVSVΔG-ZEBOV-GP product registration. AVAREF, African Vaccine Regulatory Forum; CHMP, Committee for Medicinal Products for Human Use; EMA, European Medicines Agency; EU, European Union; FDA, Food & Drug Administration; U.S., United States; WHO, World Health Organization.

The regulatory timeline for rVSVΔG-ZEBOV-GP is summarized in Figure 3.

### 3.1. Initial Product Registration and Post-Approval Changes

Registrations in Africa were initially obtained through close coordination between the European Medicines Agency (EMA; Amsterdam, The Netherlands), U.S. Food & Drug Administration (FDA; Silver Spring, MD, USA), WHO Prequalification group, African National Regulatory Authorities (NRAs), and the African Vaccine Regulatory Forum (AVAREF). WHO published the *Roadmap for introduction and roll-out of Merck rVSV-ZEBOV Ebola Virus Disease vaccine in African countries*, which detailed a path to collaborate across multiple organizations to expedite WHO prequalification and registration of MSD’s candidate vaccine in the highest risk countries in Africa [40]. Registrations in high-risk countries were pursued through the WHO collaborative registration procedure, whereby the same dossier was provided to EMA, WHO, and African countries at the same time with a target to complete the prequalification process quickly and complete registration in the highest risk countries in Africa within 90 days of WHO prequalification. Importantly, pre-alignment regarding considerations such as limitations on thermostability enabled more rapid review and approvals.

Despite the high level of collaboration, there were still challenges [41]. Five registrations were approved within 90 days of prequalification, but most registrations took longer, with several still pending more than 3 years after the dossier was first submitted, suggesting that there is still room for improvement in the process. Furthermore, lifecycle management is critical to maintain compliant registrations and product supply. An efficient and streamlined post-approval changes process is required to maintain the dossier as current. The requirements to report changes and approval timelines differ among the various NRAs. Post-approval changes need rapid and consistent approval times to maintain an efficient product supply chain.

### 3.2. Expansion of Indication to Include Pediatric and HIV-Positive Individuals

Both the U.S. FDA and EMA require manufacturers to submit plans to conduct clinical studies in the pediatric population to support the use of vaccines or drugs with the claimed indications. The PREVAC study described above [28] is included as part of the pediatric commitments to both the EMA Pediatric Investigation Plan (PIP) and U.S. FDA Pediatric Study Plan (PSP). The EMA PIP commitment further includes the ACHIV-Ebola study, which includes the evaluation of rVSVΔG-ZEBOV-GP in HIV-positive adolescents.

### 3.3. Labeling Harmonization to Facilitate Distribution of Stockpiled or Pandemic Vaccines

The initial registration of rVSVΔG-ZEBOV-GP by the EMA included bilingual English/French label and translation into 24 languages on the EMA website [24]. The initial registration by the U.S. FDA also included bilingual English/French U.S. Prescribing Information and packaging, which is precedent setting. The intent of these regulatory actions is to support flexibility (where it is not possible to predict where the next outbreak might occur), access, and distribution around the world in the event of an outbreak. The intent and goal for stockpiled vaccines contrasts with standard vaccines, where each country typically has their own specific label. Harmonized labeling is directly applicable to stockpiled or pandemic vaccines, but the concept can also be attributed to standard vaccines and medicines for efficient access and elimination of waste. However, there are aspects of local labeling that need to be addressed, such as local designated points of contact for quality and safety/pharmacovigilance reporting. For rVSVΔG-ZEBOV-GP, this need was addressed by having country-specific labels on local country marketing authorization holder and local health authority websites. Electronic labeling appears to be the best solution for the future, with a possible interim step being inclusion of both a paper label and a QR code so that end users, healthcare providers, and consumers experience the utility and ease of use of electronic labeling.

## 4. Use of rVSVΔG-ZEBOV-GP in Ebola Outbreak Response

Since 2018, there has been a series of Ebola outbreaks, focused primarily in the North Kivu and Équateur provinces of the Democratic Republic of the Congo (DRC) but also including an outbreak in Guinea in 2021. The North Kivu outbreak, which began in August 2018 and continued into June 2020, was the second largest Ebola outbreak ever recorded with 3481 cases and 2299 deaths [42]. The extensive response to this outbreak included a large vaccination effort, mainly conducted under a compassionate use clinical protocol. Ultimately, more than 300,000 at-risk individuals, including children from 6 months of age, as well as pregnant and lactating women, were vaccinated with rVSVΔG-ZEBOV-GP in the DRC [43]. In addition, tens of thousands of at-risk individuals in countries neighboring the outbreak region were also vaccinated in alignment with WHO Strategic Advisory Group of Experts on Immunization (SAGE) recommendations [44]. A recent publication based on the use of the vaccine in the DRC outbreaks suggests that rVSVΔG-ZEBOV-GP may have an important impact on preventing death due to EVD in this setting [45].

The investigational doses used for the majority of the responses were manufactured by MSD with support from the U.S. government Biomedical Advanced Research and Development Authority (BARDA) and were provided free of charge to the WHO, which spearheaded the vaccination responses in collaboration with the local and national governments of each at-risk country. In addition, various regulatory frameworks were utilized in different non-epidemic countries (e.g., France, U.S., Switzerland) to enable access to investigational doses for healthcare workers and other frontline workers being deployed to support outbreak response. The preparation and approval of appropriate protocols and availability of doses ahead of an outbreak was critical to ensuring a timely response that had the potential to impact the outbreak and is a key lesson for the development of other epidemic vaccines. Most recently, registered doses were used in the DRC beginning in late 2021 as part of outbreak response, representing an important step forward for outbreak preparedness.

## 5. Manufacturing and Stockpiling

### 5.1. General Business Model and Sustainability

The business model for vaccines that target epidemic diseases can look very different if the vaccine is intended to be used solely for outbreak response or if it is intended to be used for more prospective prophylactic vaccination. If the vaccine is used for outbreak response, the doses required will depend on the size and frequency of any outbreaks, resulting in uncertain demand. The number of outbreaks and cases in one year can vary greatly when compared with another. This can result in a situation where manufacturers cannot easily increase or decrease manufacturing levels to meet demand due to the long lead times in vaccines manufacturing. Additionally, planning for future manufacturing capacity needs in the absence of routine use and when the size, location, and frequency of outbreaks is difficult to predict is extremely challenging.

The challenge associated with uncertain demand may be addressed by the model of building stockpiles. Stockpiles allow the global public health community to build up supply in years where there may not be as much need and ensure that an adequate number of doses are available during an outbreak. They also provide manufacturers with a reliable supply signal, allowing them to plan their production cycles for stockpile build up and replenishment based on product expiry. However, if demand is restricted to a limited number of doses for stockpile procurement, this could result in expensive vaccines. When the high fixed costs of research and development, manufacturing investments, and potentially idle facilities are spread out over a limited number of doses, the result is a high cost of goods.

While stockpile models can help ensure that life-saving vaccines are available in the event of public health emergencies, significant challenges remain with this model [46]. The limited number of doses and resulting high cost of goods can pose challenges for the sustainability of government or organization stockpile budgets, while leaving manufacturers in a situation where they cannot recoup their high fixed research, development, and production expenses. New emerging models can be considered as alternatives or complements; innovative models such as advance purchase commitments or lump sum payments can help incentivize research and investment in epidemic disease vaccines and overcome the barriers for products with uncertain demand.

### 5.2. Supply/Demand Planning

The operational decisions around building and managing a stockpile require strong collaboration between the manufacturer and the procuring entity(s). The first step to establishing a stockpile is to determine the size of the stockpile that is needed. When the global stockpile for the rVSVΔG-ZEBOV-GP vaccine was proposed, MSD met with key public health stakeholders (GAVI, WHO, UNICEF) to determine the optimal stockpile size. The number of doses that would need to be drawn annually, as well as the additional doses that may be required in the event of a large-scale outbreak, were key considerations. Both MSD and public health stakeholders discussed how to best ensure public health needs would be met, while accounting for the annual financial investment that could be supported. In order to maintain the stockpile levels once established, it was critical to understand the shelf life of the doses, and the number expected to be drawn down each year. After considering these characteristics, global public health partners opted to procure and maintain a 500,000-dose stockpile for rVSVΔG-ZEBOV-GP. This global stockpile has been established under the governance of the International Coordinating Group (ICG) on Vaccine Provision [47].

While the manufacturer is responsible for ensuring timely delivery to the stockpile, other characteristics of the stockpile must be driven by governing bodies and procurement agencies. For the rVSVΔG-ZEBOV-GP stockpile, the doses are allocated by the governing body appointed to manage the stockpile. This removes the decision making of how many doses should be allocated from the manufacturer. In the event of an outbreak, public health governing bodies are best positioned to determine the vaccination strategies to be implemented and the number of doses needed to respond. In order to ensure a steady level of doses in the stockpile, deliveries to the stockpile should be metered over time to ensure that when doses are deployed, there is replenishment planned as part of regular stockpile maintenance.

Given that the number of the doses in the stockpile is a careful calculation to balance annual usage and expiry, the requirements for allocation of the stockpile doses must be well defined and transparent to ensure the balance of stockpile doses is maintained while supporting public health needs. In the case of the Ebola vaccine, there are clear SAGE guidelines which dictate when rVSVΔG-ZEBOV-GP should be used [48]. Any country that is requesting rVSVΔG-ZEBOV-GP doses must make a formal request to ICG, which is then reviewed against the guidelines for when the stockpile can be deployed. By ensuring the stockpile is only deployed for predefined situations, it tries to ensure that the stockpile dynamics of incoming doses, usage, and expiry remain balanced. Additionally, manufacturers and procurement entities must also address the operational considerations of a stockpile—storage responsibilities, legal ownership, and the financial liability of doses in the stockpile, as well as other contractual matters. Different procuring entities and governments may opt for different preferences on these operational considerations. The extent to which the global public health community can align on a set of operational norms for stockpiles can help eliminate logistical hurdles when setting up additional stockpiles in the future.

### 5.3. Supply Chain Design—Manufacturing Facility

When the West Africa Ebola outbreak of 2014–2016 was active, decisions for the long-term manufacturing of rVSVΔG-ZEBOV-GP were focused on the primary objective of speed and needed to be made in the absence of a firm demand signal. In the future, it is critical for public health partners to think prospectively about stockpile sizing for a particular medicine early in the process in order to ensure a fit for purpose and effective manufacturing and supply chain design. There can be missed opportunities, ultimately affecting product speed, lead time, and cost, such as technology application, the ability to have a multi-product facility, making the right sized capital investments, post-approval changes required, and sourcing strategies, if consideration is not given early to what is needed at the end.

Vaccine processes can be lengthy and, hence, not responsive enough to pivot quickly in an atypical demand situation or large outbreak. Due to the urgent need linked to the EBOV outbreak in West Africa, there was insufficient time to optimize the rVSVΔG-ZEBOV-GP cell expansion process and technology resulting in a lengthy, manual, and relatively expensive process/facility. Future manufacturers should consider options for manufacturing technologies to reduce the overall manufacturing time and cost or analytical technologies, such as rapid identity testing, to shorten the overall release process. Manufacturing platforms could also be used for multiple products providing the ability to have a multi-product facility and reduce idle capacity.

The regulations governing product and facility registration are continually evolving and manufacturers of future products need to determine how to set up the supply chain to balance speed with potential post-approval changes. For example, if equipment is at risk of not complying with future data integrity standards, the cost of addressing this post-registration could be more complex and expensive than addressing it from the outset. A similar concept applies to components and materials; trying to add sources can be much more complex and expensive post-approval. Given the current supply situation in the world, it is prudent to have a purposeful sourcing strategy to mitigate risk.

### 5.4. Supply Chain Design—Stockpile Enablement

At time of manufacture, it is likely unknown where a product might be delivered and administered for outbreak preparedness. This creates several areas of opportunity in customization, stockpile location, deployment, and product formulation. As highlighted earlier, the rVSVΔG-ZEBOV-GP vaccine has an image intended for pandemic preparedness, which has dual language labeling (vial label, carton, product leaflet) and is WHO prequalified for flexibility as to where the product can be utilized (see Section 3.3). There could be further opportunity to limit customization by pursuing an e-leaflet. This is an evolving industry and legislative topic driven by flexibility and environmental concerns. There are industry groups who are advocating in this space to drive change in individual country legislation. As progress is made and legislation continues to evolve, it would be opportunistic to implement.

In an outbreak, it is critical to deploy a vaccine quickly and the location of a stockpile will affect the time needed to move the material. A single stockpile may not meet the needs to deploy quickly, instead more localized stockpiles could be considered if the proper infrastructure is available. Additionally, the import/export requirements need to be evaluated for any location along with the major airports and transportation routes. As these types of vaccines could be deployed to various global locations, it is important to carefully choreograph activities. There may be multiple entities who have roles and responsibilities in the process, such as the manufacturer, the freight forwarder, and the procurer of the vaccine. There could be variables to each location which drive differences in the process, such as import requirements and whether the product is registered in the country. A recommendation is to simulate these activities ahead of time with a sampling of likely deployment locations, covering common variables to increase right first-time success in the face of an outbreak when speed is key.

As highlighted earlier, the rVSVΔG-ZEBOV-GP vaccine was rapidly implemented in Phase 3 clinical trials when the West Africa outbreak occurred. The urgency did not allow time for additional formulation development before use in the pivotal trial, resulting in the need to take forward a frozen liquid formulation that must be stored for extended periods at ≤−60 °C. To develop a second-generation product or future product, it would be worthwhile to allow additional time to develop a more thermally stable vaccine formulation that could support a refrigerated stable image and Vaccine Vial Monitoring (VVM) 7 requirement. The scale-up of a frozen liquid vaccine formulation requires careful attention to the details, particularly regarding the sensitivity to freeze and thaw conditions. The rates of freezing and thawing are critical to preserve potency of the vaccine. During the freezing and thawing processes, the product can exhibit different sensitivities to volume of fill, numbers of vials, and the positional effects during the process. Furthermore, handling of a frozen liquid product after freezing also creates significant logistical challenges. A few minutes of handling time outside the freezer and off dry ice can permit the product to warm above acceptable temperatures. Labeling and packaging before the freeze step can help minimize handling.

In-country transport conditions can differ from established product shipment conditions. Although the vaccine is normally shipped at temperatures ≤ −60 °C, additional stability data were required when the only available shippers for transfer within a country were qualified for shipment at −50 °C or lower. For some remote locations, significant logistical challenges can make frozen distribution unfeasible altogether and require transport across uneven terrain. To support transport to these locations, it was necessary to demonstrate that the prolonged (up to 7 days) agitation of the thawed formulations at 2–8 °C would not impact the product potency [49].

## 6. Authority Recommendations for rVSVΔG-ZEBOV-GP

While the drug approval process is critical, a subsequent step in ensuring product access and use is the recommendation process, separate from the approval process. For example, in the US, drugs and vaccines are approved by the FDA, but recommendations for use of vaccines come from the American Committee on Immunization Practices, which advises the CDC on final recommendations. This process, occurring after the FDA authorization, is an important subject as it ultimately determines who receives the vaccine.

Recommendations for the use of rVSVΔG-ZEBOV-GP are summarized in Table 1. WHO-SAGE has recommended the use of the vaccine for outbreak response including ring vaccination and the vaccination of healthcare and frontline workers who may be at risk [48]. The populations included in the recommendations include children from birth and pregnant and lactating women, despite the fact that these populations are not included in the approved indication. The Advisory Committee for Immunization Practices (ACIP) recommendations in the U.S. focuses on individuals at risk for occupational exposure [50,51].

## 7. Environmental Risk Assessment for rVSVΔG-ZEBOV-GP

Since rVSVΔG-ZEBOV-GP is a replication-competent genetically modified organism (GMO), environmental assessments were required to secure approvals for clinical trials and marketing authorizations. The environmental risk assessment (ERA) for rVSVΔG-ZEBOV-GP [27] was structured around guidance from both the U.S. FDA Center for Biologics Evaluation and Research and the European Union (EU) [52,53] under provisions of deliberate release of GMO) to the environment. The purpose of the ERA is to evaluate potential adverse effects and negative consequences for people other than the vaccinated persons and for the environment at large. The ERA leverages all information collected in support of the drug filing, including manufacturing (e.g., stability, decontamination), non-clinical studies (effects in animals), and clinical studies (viremia, shedding, and adverse events).

Manufacturing studies indicate that rVSVΔG-ZEBOV-GP has been shown to lose potency when held at 37 °C (1.137 log_10_ pfu/mL potency loss per day) or 25 °C (0.0790 log_10_ pfu/mL potency loss per day) and thus is expected to lose potency under ambient conditions in case of an inadvertent environmental release (unpublished data).

The potential for the transmission of rVSVΔG-ZEBOV-GP has been investigated in livestock and wild animals, including rodents (mice and hamsters), NHPs, and swine, as well as in arthropods [27,54,55,56,57,58,59]. These non-clinical studies demonstrate the safety of rVSVΔG-ZEBOV-GP for terrestrial animals, including livestock, and indicate lack of “spread” of the GMO either across species or within species. Most relevant to the ERA are the studies in swine and arthropods. In swine dosed with rVSVΔG-ZEBOV-GP, vesicular lesions consistent with vesicular stomatitis virus (VSV) infection were observed [59]. Importantly, none of the rVSVΔG-ZEBOV-GP-contact control pigs developed clinical signs of VSV infection nor had detectable rVSVΔG-ZEBOV-GP RNA or ZEBOV-GP-specific neutralizing antibodies, indicating that there was no transfer of the rVSV∆G-ZEBOV-GP virus from inoculated animals to contact control animals [59]. The study in arthropod vectors that represent actual and potential epidemic vectors of wild type VSV showed no replication in cultured cells derived from multiple arthropod species (*Anopheles* or *Aedes* mosquito, *Culicoides* biting midge, or *Lutzomyia* sand fly), nor in live *Culex* and *Aedes* mosquitoes following exposure through intrathoracic inoculation or in a high-titer infectious blood meal [55].

Viremia with rVSV∆G-ZEBOV-GP (measured by the detection of rVSVΔG-ZEBOV-GP RNA in the blood in Phase 1 studies) was common among vaccine recipients and resolved in a majority of subjects within 1 week [5,6,7,8,10]. The vaccine viral shedding of rVSV∆G-ZEBOV-GP (measured by the detection of rVSVΔG-ZEBOV-GP RNA in saliva, urine, and fluid from skin vesicles) was rare in adults [5,6,7,8,25]. In the Phase 1 study conducted in Gabon, vaccine viral shedding was assessed in adolescents and children, and it was demonstrated that they had a higher magnitude of vaccine viremia and a greater degree of vaccine viral shedding in the saliva and urine compared with reports in adults [10] but still very low levels. The person-to-person transmission of rVSV∆G-ZEBOV-GP has not been documented in any of the clinical trials.

Based on the data summarized herein, the transmission of the vaccine virus from vaccinees to other humans and to the environment represents a negligible risk. Furthermore, given the nature of the construct, if transmitted, the vaccine virus would retain its attenuated phenotype [27].

## 8. Summary

In summary, the development of the rVSVΔG-ZEBOV-GP vaccine provides an important example for approaches to testing and registration for other potential filovirus vaccines. The complete package of information that is needed to support product registration and the considerations on approaches to generate that package is complex and product dependent. However, there are significant learnings that can be applied and may help to accelerate the registration of vaccines to protect against other filovirus pathogens, such as Marburg and *Sudan ebolavirus*.

## Figures and Tables

**Table 1:** Recommendations for rVSVΔG-ZEBOV-GP.

Agency and Date of Recommendations	Recommendations
WHO SAGE [48] March 2021	Reconfirmed prior recommendation to use rVSVΔG-ZEBOV-GP for an outbreak of EBOV ○ Ring vaccination strategy recommended ○ All contacts and contacts of contacts should receive rVSVΔG-ZEBOV-GP if they have not received Ebola vaccination in the past 6 months Expanded, off-label use of rVSVΔG-ZEBOV-GP in outbreak settings for children from birth to 17 years of age and for pregnant and lactating women ○ Need to collect additional data on safety of rVSVΔG-ZEBOV-GP in these populations Did not recommend revaccination if previously vaccinated with rVSVΔG-ZEBOV-GP and not a contact or contact of contact Did not recommend preventive use of rVSVΔG-ZEBOV-GP
U.S. CDC ACIP [50] February 2020	Recommended pre-exposure rVSVΔG-ZEBOV-GP vaccination for adults aged ≥ 18 years in the U.S. at high risk of potential occupational exposure to EBOV: ○ Responding to an outbreak ○ Healthcare personnel at Ebola treatment centers ○ Laboratorians or other staff members in biosafety level 4 facilities
U.S. CDC ACIP [51] November 2021	Expansion of pre-exposure rVSVΔG-ZEBOV-GP vaccination for: ○ Healthcare personnel involved in the care and transport of patients with suspected or confirmed EVD at Ebola treatment centers ○ Laboratorians and support staff members at Laboratory Response Network facilities that handle specimens that might contain replication-competent EBOV

ACIP, Advisory Committee for Immunization Practices; CDC, Centers for Disease Control and Prevention; EBOV, *Zaire ebolavirus*; EVD, Ebola virus disease; SAGE, Strategic Advisory Group of Experts; US, United States; WHO, World Health Organization.

## Data Availability

Not applicable.
